# Supplementary material for: Spatiotemporal Transcriptomic Profiling Reveals the Dynamic Immunological Landscape of Alveolar Echinococcosis
Source: Adv Sci (Weinh). 2025 Feb 22;12(18):2405914. doi: 10.1002/advs.202405914 (PMC12079354; doi:10.1002/advs.202405914)
Supplement: Supplementary file 1 — Supporting Information [file ADVS-12-2405914-s002.docx]

Supporting Information

**Spatiotemporal transcriptomic profiling reveals the dynamic immunological landscape of alveolar echinococcosis**

Zhihua Ou, Li Li, Peidi Ren, Ting-Ting Zhou, Fan He, Jialing Chen, Huimin Cai, Xiumin Han, Yao-Dong Wu, Jiandong Li, Xiu-Rong Li, Qiming Tan, Wenhui Li, Qi Chen, Nian-Zhang Zhang, Xiuju He, Wei-Gang Chen, Yanping Zhao, Jiwen Sun, Qian Zhang, Yan-Tao Wu, Yingan Liang, Jie You, Guohai Hu, Xue-Qi Tian, Sha Liao, Bao-Quan Fu, Ao Chen, Xue-Peng Cai, Huanming Yang, Jian Wang, Xin Jin, Xun Xu, Wan-Zhong Jia,* Junhua Li,* and Hong-Bin Yan*

**
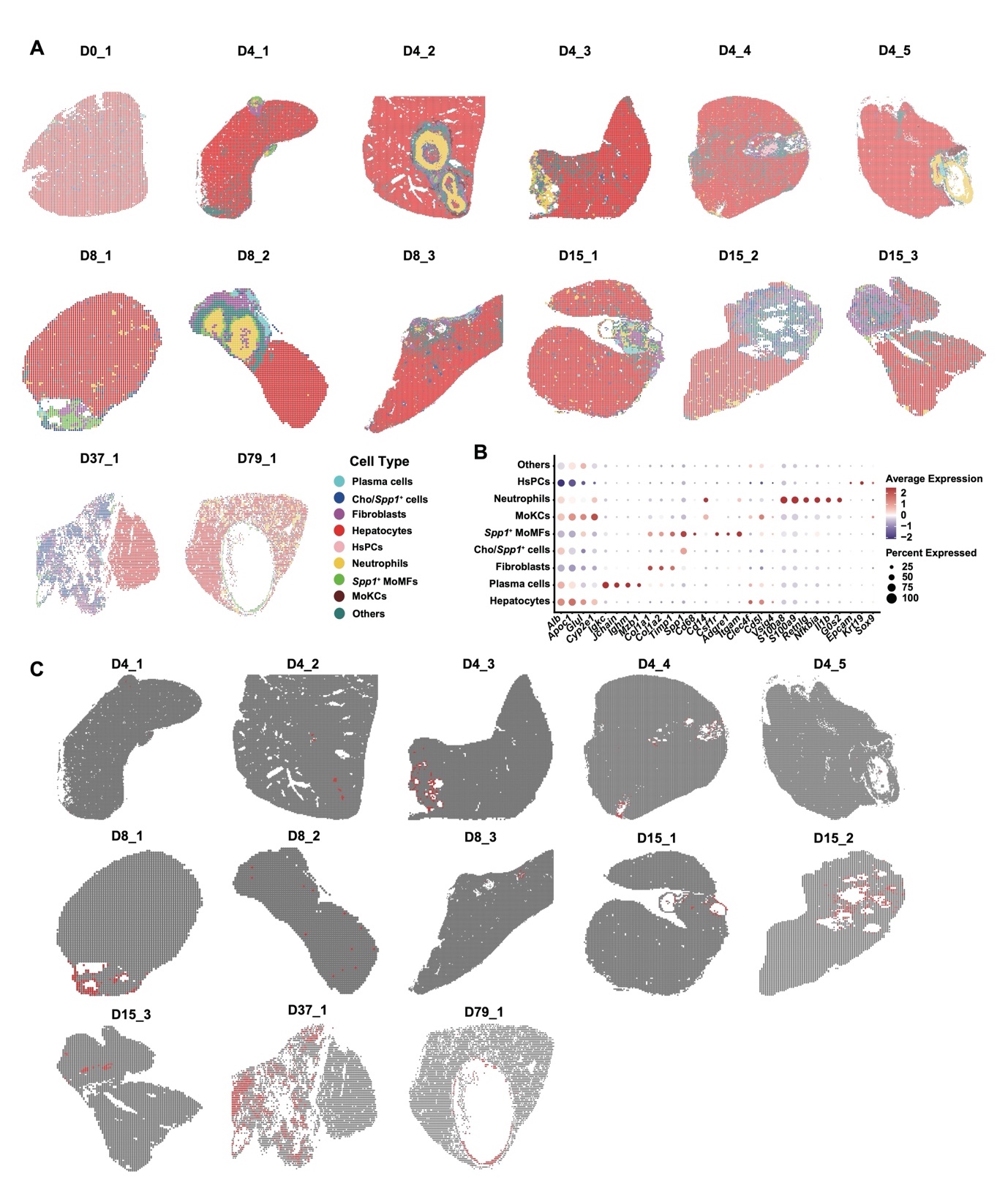
Figure S1. The spatial atlas of mouse livers with or without *Echinococcus multilocularis* infection; related to Figure 1.** (A) Annotation results of 14 Stereo-seq chips. (B) Expression of the cell type markers in cell clusters identified based on Stereo-seq data. (C) Detection of *E. multilocularis* genes in Stereo-seq chips. No parasitic genes were detected in the control liver sample.

**
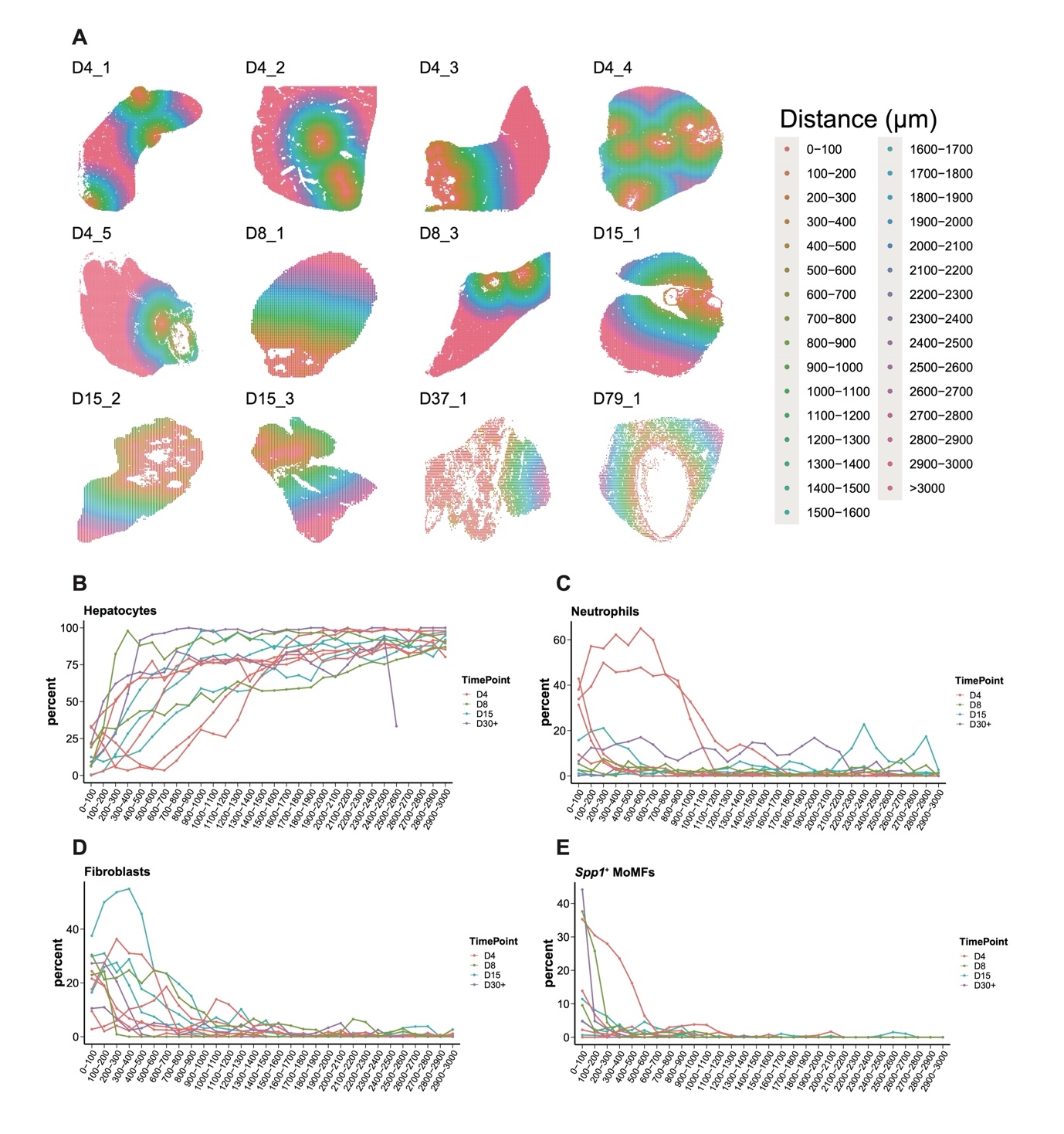
Figure S2. Spatial distribution of different cell types in mouse liver infected with *Echinococcus multilocularis*; related to Figure 1*.*** (A) Circular expansion of the tissue layers based on a unit of 200 μm, starting from the AE lesion center. (B) Distribution of hepatocytes from the center to the distal region of the AE lesion in samples of different timepoints. (C) Distribution of neutrophils from the center to the distal region of the AE lesion in samples of different timepoints. (D) Distribution of fibroblasts from the center to the distal region of the AE lesion in samples of different timepoints. (E) Distribution of *Spp1^+^* MoMFs from the center to the distal region of the AE lesions in samples of different timepoints.

**
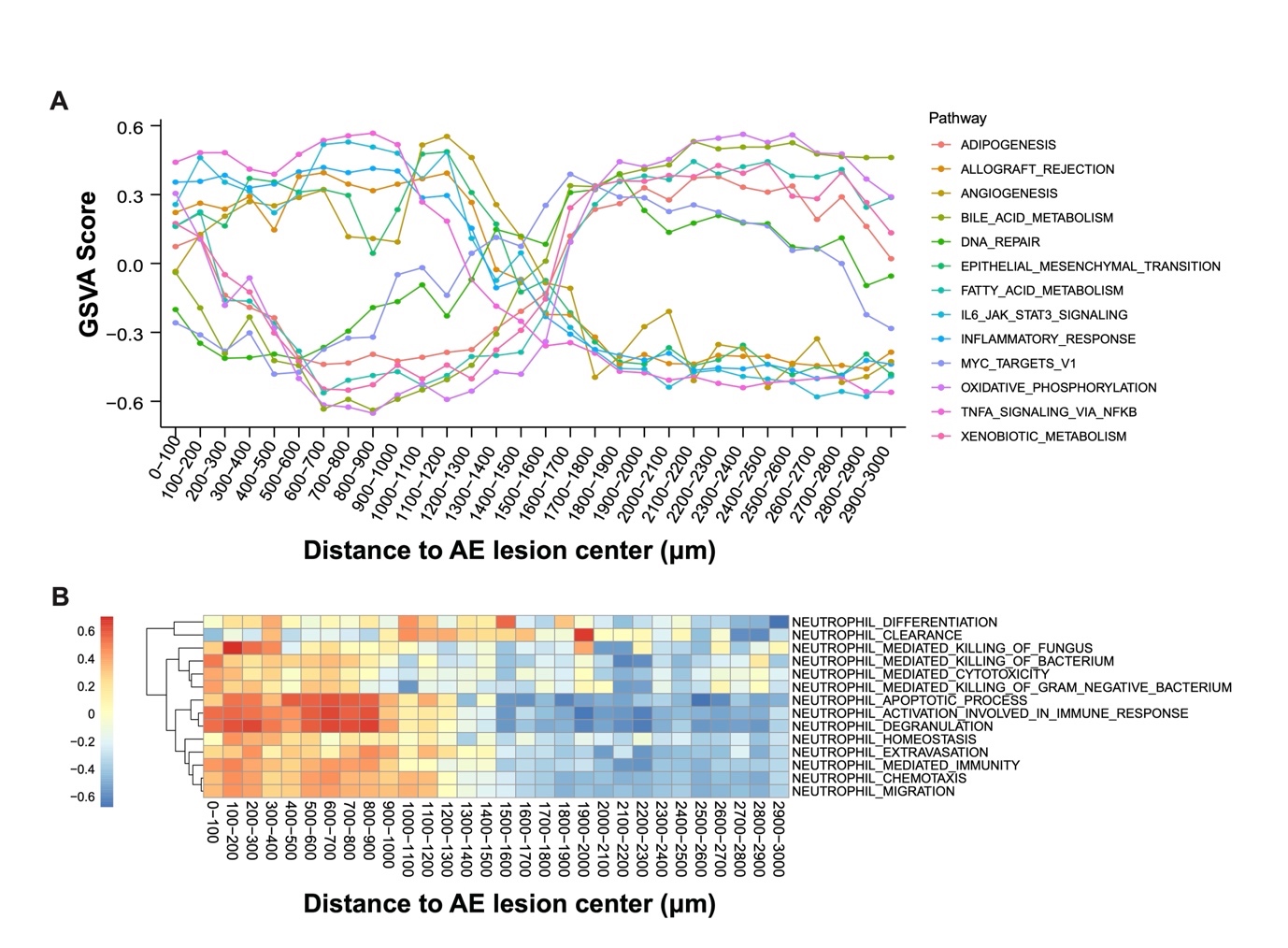
Figure S3. Spatial dynamics of biological pathways in AE lesion; related to Figure 1.** (A) GSVA scores of multiple biological pathways from the center to the distal region of the AE lesion of Sample D4_1 (4 days post infection, Sample No.1). (B) GSVA scores of neutrophil-associated pathways from the center to the distal region of the AE lesion in Sample D4_1.

**
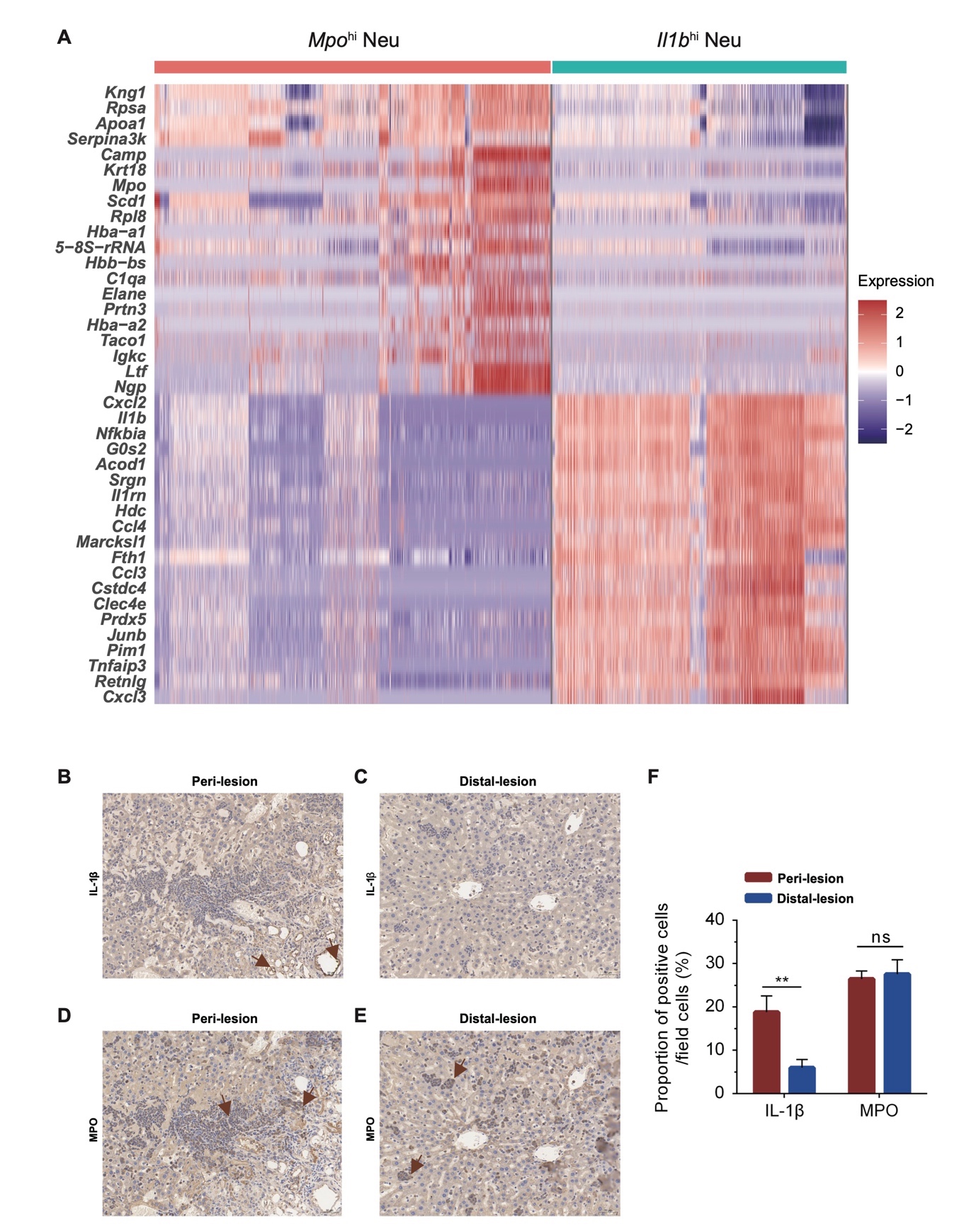
Figure S4. Identification and immunohistochemical staining of neutrophils in mouse livers infected with *E. multilocularis* (60 dpi); related to Figure 3.** (A) Heatmap of differentially expressed genes in *Mpo^hi^* Neu and *Il1b^hi^* Neu based on the Stereo-seq data. (B) Neutrophils in the peri-lesion region that were positive of IHC staining for IL-1β. (C) Neutrophils in the distal-lesion region were negative of IHC staining for IL-1β. (D) Neutrophils in the peri-lesion region that were positive of IHC staining for MPO (myeloperoxidase). (E) Neutrophils in the distal-lesion region were positive of IHC staining for MPO. (F) The percentages of positively stained cells were calculated to assess the expression of IL-1β (left) and MPO (right). Data are displayed as means ± standard deviation (SD). Statistical significance was determined using the unpaired two-tailed Student’s t test. Asterisks indicate the statistical significance between groups. *, P < 0.05; **, P < 0.01; ***, P < 0.001. ns, no statistical significance.

**
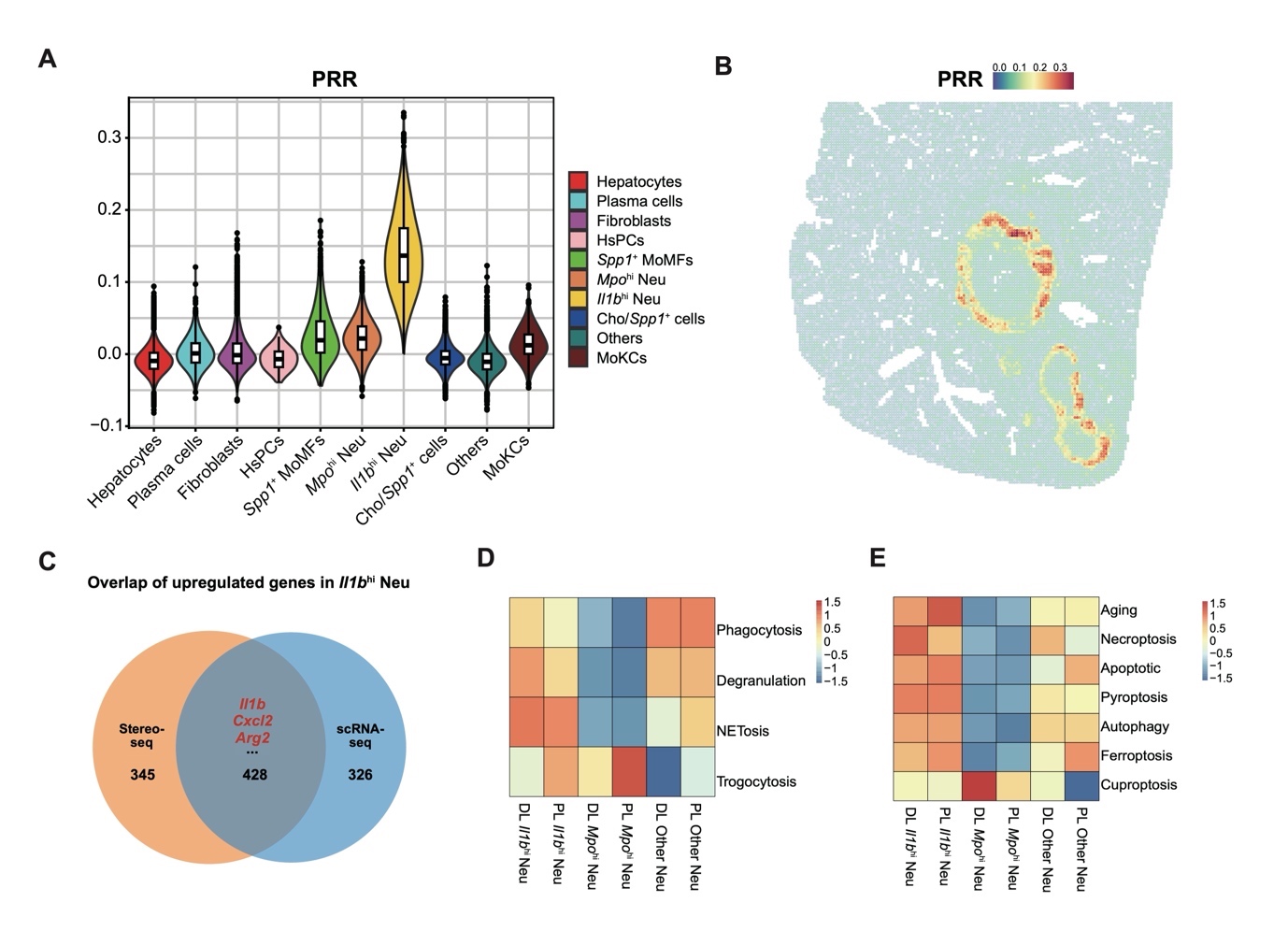
Figure S5. Transcriptional and functional heterogeneity of neutrophil subpopulations; related to Figure 3.** (A) Comparison on the pattern-recognition receptor (PRR) gene set scores between all the cell types identified in 14 Stereo-seq chips. (B) Spatial pattern of PRR gene set signatures in Sample D4_2. (C) The overlap of upregulated genes in *Il1b^hi^* Neu identified from Stereo-seq data and scRNA-seq data. (D) Heatmap showing the GSVA scores for pathogen-killing pathways of the neutrophil subgroups identified from scRNA-seq data. DL: distal-lesion; PL: peri-lesion. (E) Heatmap showing the GSVA scores for aging and cell death pathways of the neutrophil subgroups identified from scRNA-seq data.

**
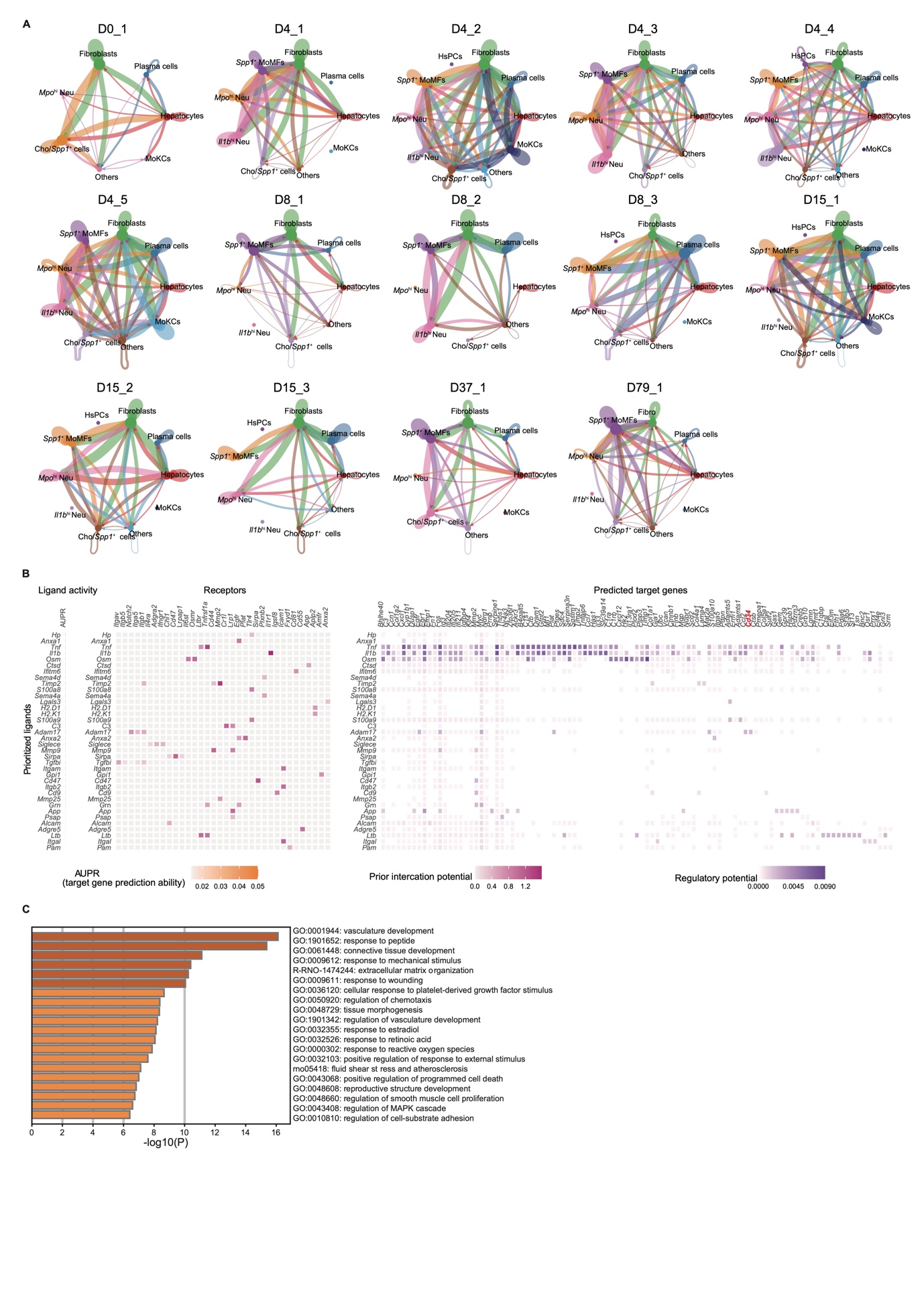
Figure S6. The cell-cell interactions between *Il1b^hi^* Neu and the other cell types; related to Figure 4.** (A) The cell-cell interaction network of 14 Stereo-seq chips. The line width represents the number of ligand-receptor pairs, and the arrow points to the receiver cell type. (B) Interactions between *Il1b^hi^* Neu and fibroblasts identified by NicheNet, based on scRNA-seq data. Top-ranked ligands on *Il1b^hi^* Neu are shown (left). Heatmap showing the ligand-receptor pairs between *Il1b^hi^* Neu and fibroblasts arranged by ligand activity (middle). Heatmap showing the targets in fibroblasts that are potentially regulated by the ligands of *Il1b^hi^* Neu (right). (C) GO enrichment of the target genes in fibroblasts that are potentially regulated by the ligands expressed by *Il1b^hi^* Neu.

**
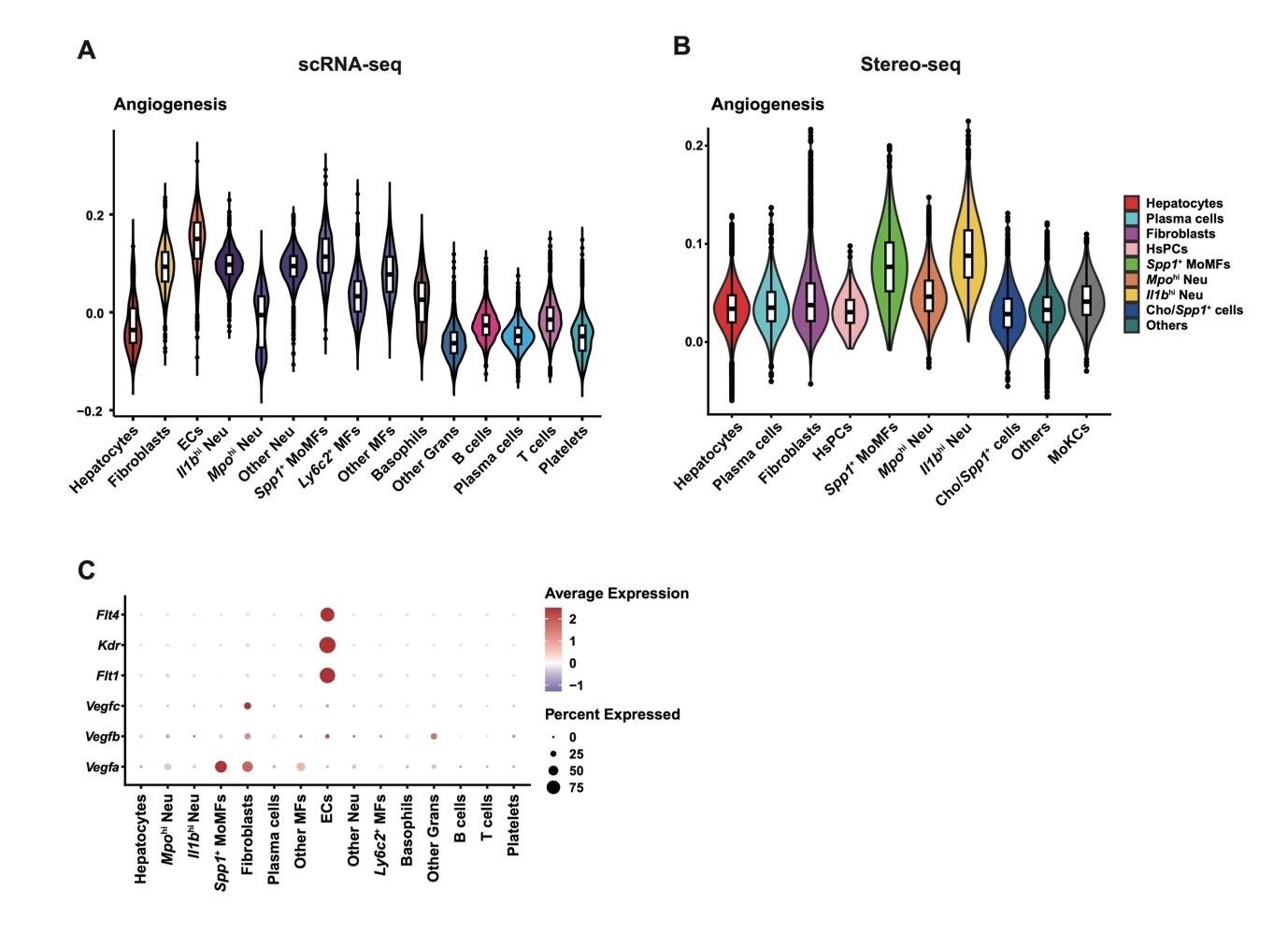
Figure S7. Angiogenesis potential of *Spp1^+^* MoMFs; related to Figure 5.** (A) Angiogenesis gene set scores of all the cell types identified from the scRNA-seq data. (B) Angiogenesis gene set scores of all the cell types identified from the 14 Stereo-seq chips. (C) Dotplot showing the gene expression levels of *Vegfa*, *Vegfb*, *Vegfc*, and their receptors (*Flt1*, *Kdr*, and *Flt4*) based on the scRNA-seq data.

**
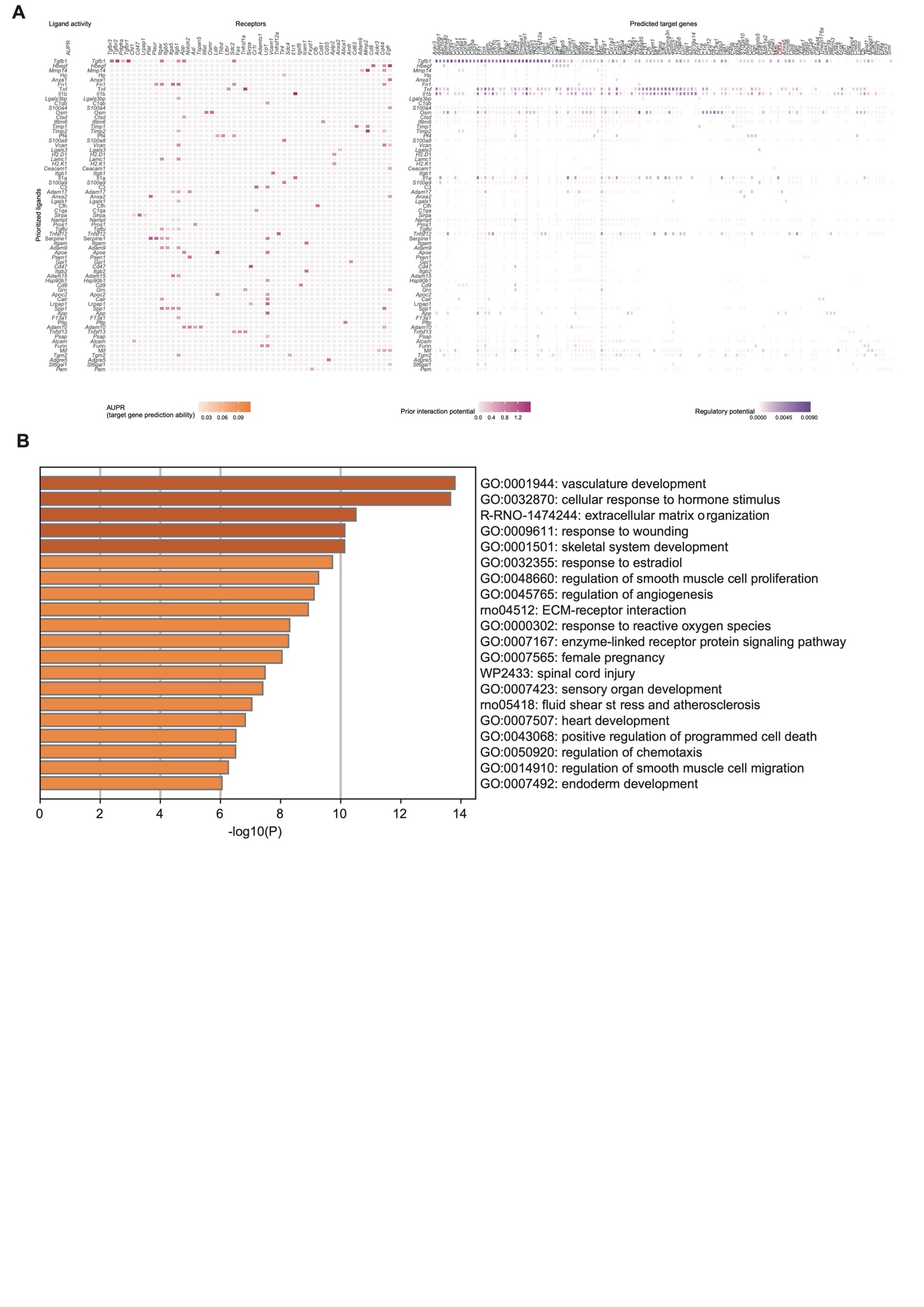
Figure S8. Ligand-receptor interactions between *Spp1^+^* MoMFs and fibroblasts identified from scRNA-seq data; related to Figure 6.** (A) Top-ranked ligands on *Spp1^+^* MoMFs are shown (left). Heatmap showing the ligand-receptor pairs between *Spp1^+^* MoMFs and fibroblasts arranged by ligand activity (middle). Heatmap showing the targets in fibroblasts that are potentially regulated by the ligands from *Spp1^+^* MoMFs (right). (B) GO enrichment of the target genes in fibroblasts that are potentially regulated by the ligands from *Spp1^+^* MoMFs.

**
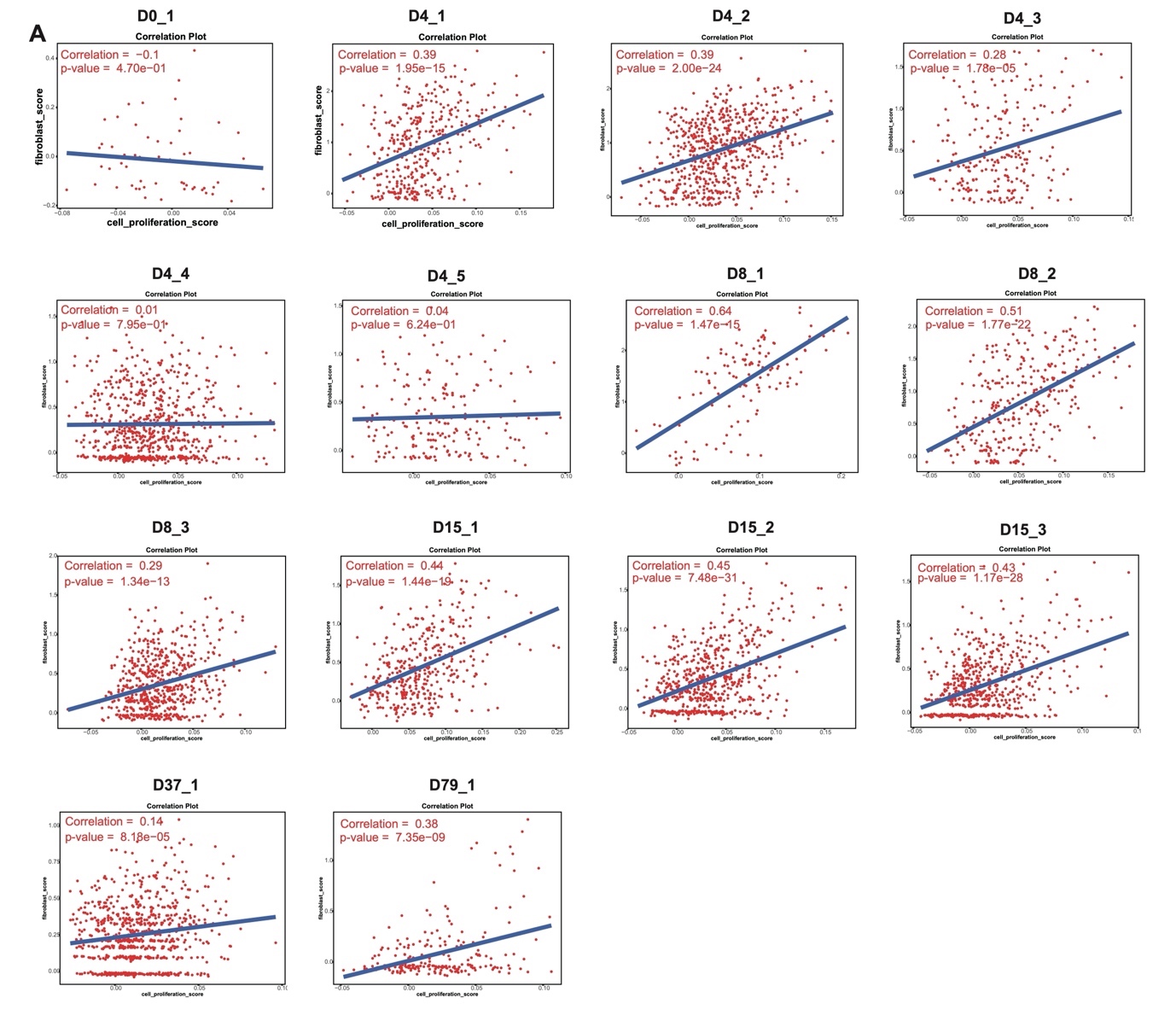
Figure S9. Assessment of the cell proliferation potential of fibroblasts; related to Figure 6.** (A) The Pearson correlation analysis of fibroblast marker genes and cell proliferation features in 14 Stereo-seq chips.
